# Efficacy of EMG- and EEG-Biofeedback in Fibromyalgia Syndrome: A Meta-Analysis and a Systematic Review of Randomized Controlled Trials

**DOI:** 10.1155/2013/962741

**Published:** 2013-09-03

**Authors:** Julia Anna Glombiewski, Kathrin Bernardy, Winfried Häuser

**Affiliations:** ^1^Department for Clinical Psychology and Psychotherapy, University of Marburg, 35032 Marburg, Germany; ^2^Department for Pain Management, Berufsgenossenschaftliches Universitätsklinikum Bergmannsheil Bochum, Ruhr University Bochum, 44789 Bochum, Germany; ^3^Department for Psychosomatic Medicine, University of Technology Munich, 80333 München, Germany

## Abstract

*Objectives*. Biofeedback (BFB) is an established intervention in the rehabilitation of headache and other pain disorders. Little is known about this treatment option for fibromyalgia syndrome (FMS). The aim of the present review is to integrate and critically evaluate the evidence regarding the efficacy of biofeedback for FMS. *Methods*. We conducted a literature search using Pubmed, clinicaltrials.gov (National Institute of Health), Cochrane Central Register of Controlled Trials, PsycINFO, SCOPUS, and manual searches. The effect size estimates were calculated using a random-effects model. *Results*. The literature search produced 123 unique citations. One hundred sixteen records were excluded. The meta-analysis included seven studies (321 patients) on EEG-Biofeedback and EMG-Biofeedback. In comparison to control groups, biofeedback (BFB) significantly reduced pain intensity with a large effect size (*g* = 0.79; 95% CI: 0.22–1.36). Subgroup analyses revealed that only EMG-BFB and not EEG-BFB significantly reduced pain intensity in comparison to control groups (*g* = 0.86; 95% CI: 0.11–1.62). BFB did not reduce sleep problems, depression, fatigue, or health-related quality of life in comparison to a control group. *Discussion*. The interpretation of the results is limited because of a lack of studies on the long-term effects of EMG-BFB in FMS. Further research should focus on the long-term efficacy of BFB in fibromyalgia and on the identification of predictors of treatment response.

## 1. Introduction

Fibromyalgia (FMS) is a chronic pain syndrome. The key symptoms of FMS are widespread pain, disturbed sleep, fatigue, and depressed mood [1, 2]. FMS affects 2–7% of the general population and predominantly affects females [3]. Psychosocial factors contribute to the development and maintenance of FMS [4], and fibromyalgia patients perceive factors such as “emotional distress” and “mental stress” as most relevant for worsening their FMS symptoms [1]. Therefore, it is important to learn more about the efficacy of psychological treatments for fibromyalgia.

Recent reviews on the efficacy of psychological interventions for fibromyalgia have reached contradictory conclusions. Thieme and Gracely [5] and Glombiewski et al. [6] found that psychological treatments, particularly cognitive-behavioural treatment (CBT), were effective methods to treat FMS, particularly with respect to the outcome of pain intensity. Bernardy et al. [7], in contrast, did not confirm these findings: CBT was found to be effective for outcomes such as coping, depressed mood, and healthcare seeking, but not pain, fatigue, or sleep. One reason for these contradictory results may have been differences in definitions of “CBT,” resulting in different study samples. Additionally, most studies on cognitive-behavioural treatment of chronic pain evaluate a “package” [8] approach that includes very different types of treatment: cognitive strategies such as attention diversion, operant techniques such as activity pacing, and respondent techniques such as relaxation or biofeedback. As van Tulder and colleagues state [8], “little is known about the actual or comparative value of different methods within cognitive-behavioural treatment,” and “it is still unclear which type of behavioural treatment is most effective, which components are necessary, and which are superfluous.” Thus, the next step is to evaluate single elements of psychological treatments for FMS, such as biofeedback.

Mind-body techniques such as progressive relaxation, hypnotherapy, and guided imagery, but not biofeedback, have been included into the definition of complementary and alternative medicine (CAM) therapies of the National Institutes of Health (NIH) (http://nccam.nih.gov/health/whatiscam/). A scheduled review on CAM-therapies in FMS will include biofeedback (Cochrane collaboration, personal communication). Biofeedback (BFB) is a very popular intervention alone or within cognitive-behavioural or multidisciplinary pain treatments. Biofeedback is a procedure in which patients' bodily responses such as muscle tension, heart rate, or skin temperature are monitored and reported to the patient through an auditory or visual modality. Various biofeedback modalities are used, electromyographic feedback (EMG-FB) being the most common for treatment of fibromyalgia. In EMG-FB, patients learn to control and to alleviate their muscle tension. Biofeedback is often called a “psychophysiological intervention,” although its change mechanisms are more psychological than physiological: it has been repeatedly demonstrated that the effectiveness of EMG biofeedback is mediated by cognitive changes, such as increases in self-efficacy and coping strategies induced through biofeedback training, rather than primarily by learned physiological control [9]. Electroencephalographic feedback (EEG-FB) is often referred to as “neurofeedback” or “EEG biofeedback.” EEG-FB records and reports back EEG waves. Patients are able to learn to influence evoked potentials, event-related potentials, slow cortical potentials, and EEG frequency components [10].

Biofeedback has been found to be beneficial in the rehabilitation of headache [11] and, in some studies, of chronic back pain [12–20] and several other pain disorders, for example, temporomandibular disorders [21, 22]. Findings from other studies on low back pain, however, show little to no improvement [23–26]. Since biofeedback is a promising intervention in the rehabilitation of headache and other pain disorders, it also might be effective in FMS. However, little is known about this treatment option for FMS, and it is not yet part of regular FMS patient care.

Thus, the aim of the present review is to integrate and critically evaluate the evidence regarding the efficacy of biofeedback for FMS.

## 2. Methods

The review was performed according to the PRISMA statement (Preferred Reporting Items for Systematic Reviews and Meta-Analyses) [27] and the recommendations of the Cochrane Collaboration [28].

### 2.1. Study Protocol

Methods of analysis and inclusion criteria were specified in advance. We used the review protocol of our systematic review on cognitive-behavioural therapies in FMS [7]. 

### 2.2. Eligibility Criteria

#### 2.2.1. Types of Interventions

Studies with any biofeedback modalities were included. Studies in which biofeedback was a part of a multicomponent therapy were excluded because it would not be possible to separate the effects of biofeedback from the additional modalities.

#### 2.2.2. Types of Studies

A randomised controlled design (RCT) comparing biofeedback with a control condition was required. In cases of multiple control groups, we defined the following order for comparison: attention placebo, treatment as usual, waiting list, and active control.

#### 2.2.3. Types of Participants

Patients diagnosed with FMS on recognized criteria (e.g., according to American College of Rheumatology (ACR) [1, 2]) were included. No age restrictions were applied.

#### 2.2.4. Types of Outcomes Measures

*Efficacy*. In order to be included, studies must have assessed at least one key domain of FMS (pain, sleep, fatigue, depression, and health-related quality of life [HRQOL]). From each trial, we selected the measure considered most appropriate for each of these four outcomes. When there was more than one measure for an outcome we gave preference to measures recommended by OMERACT [29]. 

### 2.3. Data Sources and Searches

The electronic databases screened included Pubmed, clinicaltrials.gov (National Institute of Health), Cochrane Central Register of Controlled Trials, PsycINFO, and SCOPUS (through October 12, 2012). The search strategy was adapted for each database if necessary. The search terms for Pubmed were as follows: Biofeedback [MESH] AND “Fibromyalgia”[Mesh] AND ((clinical[Title/Abstract] AND trial[Title/Abstract]) OR clinical trials[MeSH Terms] OR clinical trial[Publication Type] OR random*[Title/Abstract] OR random allocation[MeSH Terms] OR therapeutic use[MeSH Subheading]). The search keywords for clinicaltrials.gov, Cochrane Central Register of Controlled Trials, PsycINFO, and SCOPUS were as follows: (fibromyalgia AND biofeedback).

No language restrictions were applied. Only fully published articles were reviewed. In addition, reference sections of original studies, systematic reviews, and evidence-based guidelines on the management of FMS were screened manually. 

### 2.4. Study Selection

Two authors (Winfried Häuser, Kathrin Bernardy) independently screened the titles and abstracts of potentially eligible studies identified by the aforementioned search strategy. The full-text articles were then examined independently by two authors (Kathrin Bernardy, Winfried Häuser) to determine whether they met the inclusion criteria.

### 2.5. Data Collection Process

Two authors (Winfried Häuser, Kathrin Bernardy) independently extracted the data using standard extraction forms. Discrepancies were identified, and consensus was achieved by discussion. Where outcomes, means, or standard deviations were missing, attempts were made to obtain these data by contacting trial authors. Additional data were provided by three authors (see Table 1).

Data for study settings, participants, exclusion criteria, interventions, cotherapies, attendance rates, reported side effects, and outcomes sought are listed in Table 1.

### 2.6. Risk of Bias in Individual Studies

To assess the internal validity of each eligible RCT, authors (Winfried Häuser, Kathrin Bernardy) working independently determined whether the randomization, concealment of allocation, blinding of outcome assessors, and data analysis were adequate and whether intention-to-treat analysis was performed. Exclusion of patients with inflammatory diseases and psychiatric comorbidities was also assessed. The exclusion of these patients has an impact on the interpretation of the study results: since these comorbidities are very frequent in FMS the validity of the study results might be limited to a rather small group of FMS patients.

Another author (Julia Anna Glombiewski) checked the treatment settings, the means of referral to the RCT, and the demographic data of the study samples to assess whether study samples were representative (external validity) (see Table 2).

### 2.7. Summary Measures

Data entry (performed by Winfried Häuser) was checked by another author (Kathrin Bernardy). Discrepancies were resolved by consensus. Meta-analyses were conducted using RevMan Analysis software (RevMan 5.1) of the Cochrane Collaboration. Standardized mean differences (SMD) were calculated using means and standard deviations or change scores for each intervention. Examination of the combined results was performed using a random-effects model (inverse variance method), because this model is more conservative than the fixed-effects model and incorporates both within-study and between-study variances. The SMD used in Cochrane reviews is the effect size known as Hedges' (adjusted) *g*. We used Cohen's categories [30] to evaluate the magnitude of the effect size, calculated by SMD, with *g* > 0.2–0.5 = small effect size, *g* > 0.5–0.8 = medium effect size, and *g* > 0.8 = large effect size.

Heterogeneity was tested using the *I* ^2^ statistic, with *I*² values over 50% indicating strong heterogeneity.

### 2.8. Risk of Bias across Studies

Potential publication bias (i.e., the association of publication probability with the statistical significance of study results) was investigated using the Egger test, in which the standardized effect size (effect size calculated by standard error) is regressed on precision (inverse of standard error) [31]. The intercept value is an estimate of asymmetry of the funnel plot. Positive values (>0) indicate higher levels of effect size in studies with smaller sample sizes. Moreover, Begg's rank correlation test was performed using *P* < 0.05 as the criterion for significance [32].

### 2.9. Additional Analyses****

*Subgroup Analysis*. Provided that at least two studies were available, subgroup analyses were prespecified for type of BFB. These subgroup analyses were also used to examine potential sources of clinical heterogeneity.

## 3. Results

### 3.1. Search Results

**Figure 1:**
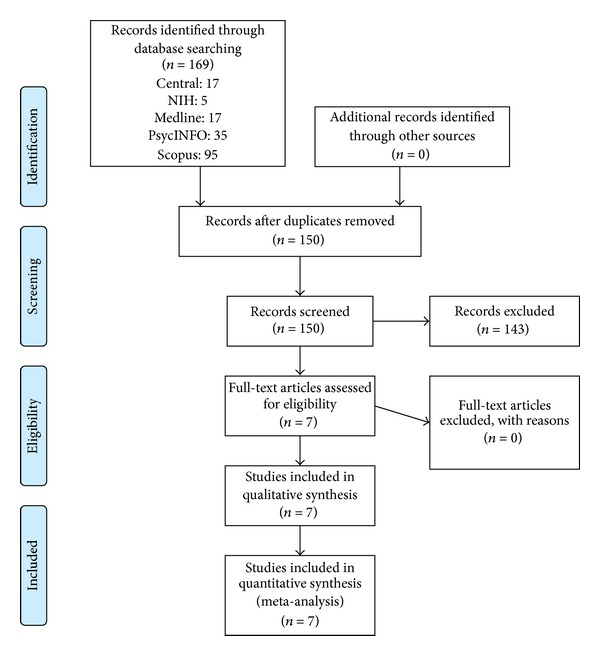
PRISMA flow diagram.

After removing duplicates, the literature search produced 150 unique citations. Through screening, 143 records were excluded. Seven full-text articles were assessed for eligibility, and none were excluded. These seven studies were included in the meta-analysis (see Figure 1).

### 3.2. Study Characteristics

#### 3.2.1. Setting, Referral, and Diagnostic Criteria

Study characteristics are described in Table 1. Three studies were conducted in North America (USA), three in Europe (Turkey, Italy, and the Netherlands), and one in Asia (India). Patients were recruited via central registers, in hospitals, private practices, self-help groups, and newspaper advertisements. All studies were conducted in an outpatient setting. Two studies included patients with inflammatory diseases [33, 34]. Two studies included patients with psychiatric comorbidities [33, 35], while two other studies specifically excluded such patients [34, 36]. With one exception, FMS was diagnosed using the criteria of the American College of Rheumatology [37].

#### 3.2.2. Participants

Patient characteristics are summarized in Table 1. 321 patients were included in the meta-analysis. The range of the mean ages of participants in the studies was 32–57 years. The participants were predominantly female and white.

#### 3.2.3. Interventions

The number of sessions varied between 6 and 22 sessions. The length of sessions was not always exactly reported and varied between 30 minutes and 3 hours. The attendance rates were not reported in most of the included studies. 

Three studies included EEG-BFB (see Table 1). Kravitz et al. [36] investigated the use of “LENS,” a low-intensity neurofeedback system. According to the authors, this method uses “a combination of a conventional EEG-BFB and subthreshold photic stimulation in order to change EEG patterns” (page 43). With LENS, patients do not consciously learn control over the brain activity. Instead, the brain wave changes are a result of the interaction of the brain and resonant changes in the feedback pulses. The brain is supposed to develop a wider range of responses to different sensory inputs. The patients in the Kravitz et al. study [36] received 22 individual sessions of LENS EEG-BFB. Nelson et al. [38] also investigated LENS EEG-BFB delivered in 22 individual sessions. Kayiran et al. [39] investigated sensimotor rhythm (SMR) training, an EEG-BFB procedure that aims to facilitate thalamic inhibitory mechanisms. SMR training is supposed to increase P300 amplitudes. In contrast to LENS, patients doing SMR training receive feedback (e.g., visual) and learn to react to that by changing the brain waves. The length of treatment was 20 sessions.

Four studies offered EMG-BFB (see Table 1). In the Babu et al. study [34] EMG electrodes were applied to the forearm extensors, upper trapezius (neck), and frontalis (forehead). In 6 individual sessions, patients learned with help of visual and auditory feedback to relax these muscles. In the Buckelew et al. study [33] patients were taught cognitive and muscular relaxation strategies, partly supported by EMG-BFB of the trapezius. The length of treatment was six rather long (1.5–3.5 hours) individual sessions. In the Ferraccioli et al. study [35] the EMG electrodes were applied to the frontalis muscle. Patients were trained in progressive muscle relaxation while receiving auditory feedback on their muscle tension during 15 sessions. The same procedure was applied in the van Santen et al. study [40], with a treatment length of 16 sessions.

In four studies the controls received Sham Biofeedback (the feedback that patients received was not correlated with actual muscle tension or brain activity). Other control groups received attention-placebo treatment, a serotonin-reuptake inhibitor, and treatment as usual. 

#### 3.2.4. Outcomes

The most frequently used instrument for the outcome measures pain, sleep, and fatigue was a visual analogue scale (VAS) (see Table 1). Sleep was assessed using the Medical Outcome Study Sleep Scale in one case. Depression was assessed using a VAS, the Beck Depression Inventory, the Center for Epidemiological Studies Depression Scale, and the Patient Health Questionnaire. HRQOL was assessed by the total score of the Fibromyalgia Impact Questionnaire in four cases and by the Sickness Impact Profile in one case.

Four studies performed follow-ups between 1 week and 6 months.

### 3.3. Risk of Bias within Studies

Most studies were of poor methodological quality (see Table 2). In all studies at least two of six important criteria were not reported. In one study three of six predefined methodological quality criteria were clearly fulfilled; in all other studies two or fewer criteria were fulfilled. The risk of bias is therefore unclear to high for each study.

### 3.4. Synthesis of Results

The effect sizes (Hedges' *g*) for all outcome measures (“Pre-post” and “Pre-follow-up” for uncontrolled effect sizes and “Controlled” and “Follow-up controlled” for controlled effect sizes) are presented in Table 3.

Based on Cohen's categories, the uncontrolled pre-post effects were large for pain intensity, fatigue, and HRQL. In comparison to control groups, BFB also significantly reduced pain intensity with a large effect size. BFB did not reduce sleep problems, depression, fatigue, or HRQL in the short term in comparison to control groups. It also did not reduce pain intensity, sleep problems, depression, fatigue, or HRQL in the long term.

Subgroup analyses revealed that only EMG-BFB, and not EEG-BFB, significantly reduced pain intensity in comparison to control groups.

Three studies reported on adverse events. One study mentioned that no adverse events occurred. Two other studies reported “stress” due to EMG-BFB, and one study reported a variety of side effects, such as headache, fatigue, and sleep problems due to EEG-BFB (see Table 1).

The dropout rate in the BFB conditions across the studies was 12%. The relative risk in comparison to the control group was 1.3 (95% CI: .67–2.49), *P* = 0.44. Thus, the dropout rate in the BFB groups was not significantly higher than the dropout rate in the control groups.

### 3.5. Risk of Bias across Studies

There was substantial heterogeneity for all computed effect sizes (see Table 3). The heterogeneity (measured with *I* ^2^) was still above 50% after subgroup analysis.

*Publication Bias*. Begg's and Egger's tests indicated a publication bias. Egger's intercept was −5.2 (*P* two tailed = 0.07), and Begg's Kendall-tau without continuity correction was −13.0 (*P* two-tailed = 0.05).

## 4. Discussion

This first meta-analysis of the efficacy of biofeedback in fibromyalgia found that EMG-BFB was effective for a (short-term) reduction of pain intensity in fibromyalgia patients with a large effect size. The sample was representative of the FMS population. The methodological quality of most of the included studies was poor, limiting the credibility of the results of this meta-analysis.

This result is promising; however, the long-term effects of EMG-BFB on pain intensity are unclear due to the small number of studies that included follow-up assessments. Although significant, the pre-post effect is based on a small number of quite heterogeneous studies. Neither EEG nor EMG biofeedback was effective in comparison to control groups at reducing depression, sleep problems, and fatigue or at increasing health-related quality of life. Considering the complexity of FMS psychopathology, it is questionable whether short-term pain reduction alone leads to significant change for better. Long-term data and more studies including a variety of outcome measures [29] are needed in this area. In addition, as all of the studies included in the meta-analysis investigated BFB treatment of FMS on an outpatient basis, it is possible that our findings may not be generalized to inpatient settings.

Our findings are noteworthy because no other meta-analysis on psychological or multicomponent treatments of fibromyalgia has reported comparably large effects on pain intensity [6, 41]. Thus, the data suggest that EMG-BFB might reduce pain intensity more successfully than other psychological or multicomponent programs. Accordingly, knowledge of the mechanisms of this biofeedback-induced pain reduction in fibromyalgia patients is critical for developing more successful treatment programs for FMS. Unfortunately, within the present meta-analysis, we were not able to perform mediator or moderator analyses. It is known from research on headaches that improvements in headache activity are correlated with increases in self-efficacy induced by biofeedback training and not with changes in EMG activity [42]. These results suggest that cognitive changes rather than physiological changes may underlie the effectiveness of biofeedback therapies. It is also possible that cognitive changes may be the most effective mechanism of biofeedback treatment for FMS, since fibromyalgia patients suffer from a high perceived uncontrollability of pain [1]. Thieme and colleagues [43] showed that FMS patients have low baseline EMG levels and, in contrast to low back pain patients [44], do not show unusually enhanced muscle tension as a reaction to stress. Accordingly, it is possible that there is no need to train muscle relaxation in FMS and that pain reduction occurs via other mechanisms. However, with the EEG-BFB modality “LENS” used in three of four EEG-BFB studies, patients do not consciously learn control over brain activity; therefore, it is unlikely that they experienced a sense of control over their symptoms. This may be one explanation for the lack of efficacy of EEG-BFB in comparison to control groups. In addition, one of the studies on LENS was criticised for significant flaws in the hardware used in the study [45].

The role of BFB treatment in the context of other treatments for FMS is important to consider. There is an important difference between the BFB treatment of chronic headache and BFB treatment of chronic low back pain; with chronic headache, BFB can be the sole therapy, while with chronic back pain BFB should be combined with other modalities. In FMS, BFB should also not be delivered as monotherapy. First, FMS is a complex syndrome, and its many symptoms are not addressed comprehensively by BFB. Second, there are other effective treatment options for FMS, such as aerobic exercise therapy [46–48] or pharmacotherapy [49, 50]. In one of the included studies [40], exercise treatment was superior to BFB at reducing pain intensity and fatigue. Another study [33] found that a combination of BFB and exercise therapy was more successful than BFB alone at follow-up. In addition, since BFB may change cognitive rather than physiological processes, it should be delivered by professionals trained in psychological interventions. Treatment providers should be able to embed cognitive interventions (e.g., education about chronic pain and enhancement of self-efficacy through cognitive restructuring) into BFB treatment. Ideally, EMG-BFB should be a part of a multimodal treatment program for FMS.

A strength of our study is a thorough literature search, which resulted in the identification of additional relevant studies on biofeedback compared to previous reviews on psychological treatments for FMS. Additional strengths are the inclusion only of RCTs and the computation of controlled effect sizes for several different outcome measures, as recommended by IMMPACT [51]. The study was conducted according to the methods used in Cochrane Reviews on chronic pain [28]. We followed recent recommendations on methods of meta-analysis [27].

One limitation of meta-analyses in general is the fact that a meta-analysis is strongly influenced by factors such as study selection criteria, the quality of the included studies, and expectancy effects [52]. However, as noted by Hunter and Schmidt [53], methodological weaknesses of included studies lead to more conservative effect size estimates. In order to limit possible biases, we analyzed the effect sizes using a random-effects model and checked for potential publication bias. The heterogeneity analysis indicated that reported effects might be partly explained by specific study characteristics. Unfortunately, we could not address this problem because there were too few studies to perform moderator analyses. Although efforts were made to obtain missing data from authors, this was not successful in every case. Moreover, there is a risk of publication bias. Last but not least, we integrate a heterogeneous mixture of treatment approaches and methods. Therefore, the validity of the integrated effect sizes is limited.

Adverse events are rarely assessed in behavioral research. In two of three studies that reported adverse events, up to 74% of patients reported side effects. Thus, we strongly recommend a systematic assessment of side effects in further BFB research and treatment. Additionally, we suggest assessing responder rates (defined as 30% pain intensity reduction) as a primary outcome [54].

Based on the results on pain intensity reduction, we recommend incorporating EMG-BFB into psychological or multidisciplinary fibromyalgia treatment programs.

Further research should focus on the long-term efficacy of BFB in FMS. Experimental research has shown low heart rate and high skin conductance levels in FMS patients as a reaction to stress [43]; these findings suggest that future research should investigate BFB modalities such as skin conductance and heart rate variability. In order to further follow the question whether cognitive or physiological changes underlie the effectiveness of BFB therapies we recommend including CBT as a control group and investigating mechanisms of change in both BFB and CBT groups.

Finally, it is important to identify moderators (e.g., pain-related self-efficacy) and predictors of treatment response as well as mechanisms of change. Any future trials should address the deficiencies noted in the quality assessment.

## Figures and Tables

**Table 1:** Main study characteristics.

Author; country; year; setting; referral or recruitment	Mean age; female %; race %	Diagnosis of FMS^1^	Study population	Treatment group	Control group	Both groups	Outcomes used for meta-analysis latest follow up
			*N* screened/*N* randomized (%); *N* completing therapy (%)	Type of treatment; duration of treatment; *N* at baseline/*N* completing therapy (%)	Type of treatment; duration of treatment; *N* at baseline/*N* completing therapy (%)	Comedication allowed; attendance rates (all sessions); side effects; drop out due to side effects in treatment group	
Babu et al.; India; 2007; outpatient; not reported [34]	39; 70%; Not reported	ACR^2^ criteria	Not reported/30; 30 (100%)	EMG-BFB^3^; 6 individual sessions of 45 minutes; 15/15 (100%)	Sham EMG-BFB; 6 individual sessions of 45 minutes; 15/15 (100%)	not reported; not reported; not reported; not reported	Pain VAS^4^ 0–10* Sleep VAS 0–10** Fatigue VAS 0–10** Depression VAS 0–10** HRQOL^5^ FIQ^6^ Total VAS 0–80*No follow-up

Buckelew et al.; ^7^USA; 1998; outpatient; university hospital, private practice [33]	44; 90.8%; not reported	Yunus criteria	240/119 (49.6%); 109 (91%)	EMG-BFB and relaxation; 6-week individual treatment once a week for 1.5–3 hours; 29/27 (93.1%)	Attention control; 6-week individual treatment once a week for 1.5–3 hours; 30/28 (93.3%)	Yes not reported; not reported; not reported	Pain VAS 0–10* Sleep VAS 0–10* Fatigue NA^8^ Depression CES^9∗^ HRQOL NA 24 months

Ferraccioli et al.; Italy; 1987; outpatient; consecutive patients at an outpatient clinic [35]	57; 100%; not reported	Not exactly specified	Not reported/12; 12/12 (100%)	EMG-BFB; 15 sessions, two sessions a week; 6/6 (100%)	Sham EMG-BFB; 15 sessions; 6/6 (100%)	Allowed; not reported; not reported; none	Pain VAS 0–10 Sleep NA Fatigue NA Fatigue NA Depression NA HRQOL NA No follow-up

Kayiran et al.; Turkey; 2010; outpatient; consecutive patients at an outpatient clinic [39]	32; 100%; not reported	ACR criteria	Not reported/40; 36 completing the first follow-up (90%)	EEG-BFB^10^; 20 individual sessions of 30 minutes; 20/18 completing the first follow-up (90%)	Escitalopram (an SSRI^11^) 10 mg/day; 8 weeks; 20/18 completing the first follow-up (90%)	not allowed; not reported; not reported; not reported	Pain VAS 0–10 Sleep NA Fatigue VAS 0–10 Depression BDI^12^ (NRS^13^ 0–63) HRQOL FIQ Total VAS 0–80 4 months

Kravitz et al.; USA; 2006; outpatient; at two private practices and via advertisement and self-help groups [36]	46.9; 92%; 92% white	ACR criteria	159/64 (40.3%); 59 (92.2%)	EEG-BFB; 22 sessions; 33/31 (93, 9%)	Sham EEG-BFB; 22 sessions; 31/28 (90.3%)	Morphines, SSRIs, and Benzodiazepines not allowed, other medication allowed; 74% reported side effects of EEG-BFB, e.g. fatigue, headache and sleep problems; not reported	Pain VAS 0–10 Sleep VAS 0–10 Fatigue VAS 0–10 Depression VAS 0–10 HRQOL FIQ Total VAS 0–80** 1 week

Nelson et al.; USA; 2010; outpatient; FMS database and advertisement [38]	51.6; 100%; 88.2% white	ACR criteria	82/42 (51.2%); 34 (81%)	EEG-BFB; 22 sessions; 21/17 (81%)	Sham EEG-BFB; 22 sessions; 21/17 (81%)	Allowed; not reported; none; none	Pain VAS 0–10* Fatigue VAS 0–10* Sleep MOS Sleep^14^ NRS 0–100 Depression PHQ 9^15^ HRQOL FIQ Total VAS 0–80 6 months

Van Tulder et al.; the Netherlands; 2001; outpatient; central registry [8]	46.2^16^; 100%; not reported	ACR criteria	268/143^17^ (53.4%); 118 (82.5%)	EMG-BFB; 16 sessions; 50/43 (86%)	Treatment as usual; consecutive medical care; 29/28 (96.55%)	Allowed; 88% of patients attended 67% of BFB sessions; 2 patients reported stress due to BFB^18^; 2 patients	Pain VAS 0–10 Sleep NA Fatigue VAS 0–100 Depression NA HRQOL SIP^19^ Total (NRS 0–100) 6 months

^1^FMS: fibromyalgia syndrome; ^2^ACR: American College of Rheumatology; ^3^EMG-BFB: electromyography biofeedback; ^4^VAS: visual analogue scale; ^5^HRQOL: health-related quality of life; ^6^FIQ: Fibromyalgia Impact questionnaire; ^7^The study included 4 different conditions. The analysis was performed with two of these conditions (Biofeedback and attention control); the numbers represent all four groups; ^8^NA: not assessed; ^9^CES: center for Epidemiological Studies Depression Scale; ^10^EEG-BFB: electroencephalography biofeedback (Neurofeedback); ^11^SSRI: selective serotonin reuptake inhibitor (an antidepressant); ^12^BDI: Beck Depression Inventory; ^13^NRS: Numeric Rating Scale; ^14^MOS: Medical Outcomes Study Sleep Scale; ^15^PHQ 9: Patient Health Questionnaire, ^16^In the BFB group; ^17^the study included 3 different conditions. The analysis was performed with two of these conditions (biofeedback and treatment as usual control); the numbers represent all three groups. ^18^BFB: biofeedback; ^19^SIP: sickness impact profile.

Note: Studies are presented in alphabetical order.

*Data provided on request.

**Data not provided on request.

**Table 2:** Methodological quality.

Study	Adequate randomization	Adequate allocation	Blinding of assessor	Intention to treat analysis
Babu et al. [34]	NR^1^	NR	NR	Yes
Buckelew et al. [33]	NR	Yes	NR	No
Ferraccioli et al. [35]	No	NR	NR	Yes
Kayiran et al. [39]	NR	NR	Yes	NR
Kravitz et al. [36]	Yes	NR	NR	Yes
Nelson et al. [38]	NR	Yes	NR	No
Van Tulder et al. [8]	NR	NR	NR	No

^1^Not reported.

**Table 3:** Effect sizes for all outcome measures.

Outcome	Type of effect	*k *(*n*)^1^	*g* ^2^	95% CI^3^	*I* ^2^	*P *
Pain intensity	Pre-post^4^	7 (167)	**1.92**	0.93–2.9	92	0.000
Pain intensity	Pre-follow-up^5^	3 (65)	1.92	−0.13–3.98	95	0.07
Pain intensity	Controlled^6^	7 (289)	**0.79**	0.22–1.36	79	0.006
Pain intensity	Follow-up controlled^7^	2 (86)	0.86	−1.25–2.98	94	0.42
Sleep problems	Pre-post	2 (92)	0.41	−0.0–0.83	0	0.05
Sleep problems	Pre-follow-up	—	**—**	—	—	—
Sleep problems	Controlled	2 (87)	0.23	−0.20–0.65	0	0.29
Sleep problems	Follow-up controlled	—	—	—	—	—
Depression	Pre-post	4 (91)	0.72	−0.15–1.59	87	0.11
Depression	Pre-follow-up	3 (71)	0.71	−0.34–1.76	88	0.18
Depression	Controlled	4 (181)	0.37	−0.44–1.18	85	0.37
Depression	Follow-up controlled	3 (120)	0.8	−0.51–2.11	91	0.23
Fatigue	Pre-post	4 (117)	**1.65**	0.28–3.03	94	0.02
Fatigue	Pre-follow-up	3 (65)	1.78	−0.25–3.81	95	0.09
Fatigue	Controlled	4 (163)	0.38	−0.46–1.08	85	0.43
Fatigue	Follow-up controlled	—	—	—	—	—
HRQOL	Pre-post	4 (106)	**2.05**	0.40–3.69	95	0.01
HRQOL	Pre-follow-up	2 (34)	6.5	−5.7–18.7	98	0.3
HRQOL	Controlled	4 (163)	0.62	−0.77–2.02	93	0.38
HRQOL	Follow-up controlled	2 (68)	0.252	−2.94–7.98	97	0.37

Comparison of EMG-BFB^8^ and EEG-BFB^9^						
Pain intensity EMG^10^	Pre-post	4 (86)	**1.46**	0.36–2.36	88	0.009
Pain intensity EMG	Pre-follow-up	—	—	—	—	—
Pain intensity EMG	Controlled	4 (162)	**0.86**	0.11–1.62	76	0.03
Pain intensity EMG	Follow-up controlled	—	**—**	—	—	—
Pain intensity EEG^11^	Pre-post	3 (65)	**2.8**	0.41–5.19	96	0.02
Pain intensity EEG	Pre-follow-up	—	**—**	—	—	—
Pain intensity EEG	Controlled	3 (127)	0.71	−0.37–1.8	86	0.2
Pain intensity EEG	Follow-up controlled	—	—	—	—	—

^1^ *k*: number of studies in the analysis, *n*: number of patients in the analysis.

^2^Effect size, Hedges *g* (significant effect sizes are marked in bold text).

^3^CI: confidence interval.

^4^Pre-post: effect size was computed for the difference of means between pretreatment and posttreatment (short-term efficacy).

^5^Pre-follow-up: effect size was computed for the difference of means between pretreatment and the longest available follow-up (long-term efficacy).

^6^Controlled: Effect size was computed for the group mean at posttreatment in comparison to a control group mean at posttreatment (short-term efficacy, controlled).

^7^Follow-up controlled: effect size was computed for the group mean at the latest follow-up in comparison to a control group mean at posttreatment (long-term efficacy, controlled).

^8^EMG BFB: electromyography biofeedback.

^9^EEG BFB: electroencephalogram biofeedback.

^10^EMG: subgroup of studies using EMG BFB.

^11^EEG: subgroup of studies using EEG BFB.
